# Origin and evolution of the deep thermochemical structure beneath Eurasia

**DOI:** 10.1038/ncomms14164

**Published:** 2017-01-18

**Authors:** N. Flament, S. Williams, R. D. Müller, M. Gurnis, D. J. Bower

**Affiliations:** 1 EarthByte Group, School of Geosciences, Madsen Building F09, University of Sydney, Sydney, New South Wales 2006, Australia; 2 Seismological Laboratory, California Institute of Technology, Pasadena, California 91125, USA; 3 Institute of Geophysics, Department of Earth Sciences, ETH Zürich, Sonneggstrasse 5, 8092 Zürich, Switzerland

## Abstract

A unique structure in the Earth's lowermost mantle, the Perm Anomaly, was recently identified beneath Eurasia. It seismologically resembles the large low-shear velocity provinces (LLSVPs) under Africa and the Pacific, but is much smaller. This challenges the current understanding of the evolution of the plate–mantle system in which plumes rise from the edges of the two LLSVPs, spatially fixed in time. New models of mantle flow over the last 230 million years reproduce the present-day structure of the lower mantle, and show a Perm-like anomaly. The anomaly formed in isolation within a closed subduction network ∼22,000 km in circumference prior to 150 million years ago before migrating ∼1,500 km westward at an average rate of 1 cm year^−1^, indicating a greater mobility of deep mantle structures than previously recognized. We hypothesize that the mobile Perm Anomaly could be linked to the Emeishan volcanics, in contrast to the previously proposed Siberian Traps.

## Abstract (web summary)

The Perm anomaly is found in the lower mantle beneath Eurasia, but how this structure formed has remained unclear. Here, the authors show that the anomaly has been mobile since it formed in isolation within a closed subduction network and propose that the anomaly is linked to the Emeishan volcanics.

The long-wavelength structure of Earth's lowermost mantle is characterized by two large low-shear velocity provinces (LLSVPs) under Africa and the Pacific, ∼15,000 km (Fig. 1a) in diameter and 500–1,000 km high^1^,^2^. In addition, a single, spatially small (∼<1,000 km in diameter, ∼500 km high) deep mantle structure named the ‘Perm Anomaly' was recently identified through seismic tomography^3^. The discovery of the Perm Anomaly poses fundamental questions about its dynamic relationship with the much larger LLSVPs, its uniqueness, its age and the formation of lower mantle structures in general. The structure of the lower mantle is important for reconstructions of the plate–mantle system^4^,^5^,^6^,^7^,^8^ in deep geological time because the reconstructed locations of most large igneous provinces (LIPs) and kimberlites over the past 320 million years (Myr) correlate with the edges of present-day LLSVPs, leading to the concept of a plume generation zone at LLSVP boundaries^6^. This concept has been used to build a model of absolute motion of tectonic plates over the Phanerozoic (past 540 Myr)^8^, under the assumptions that LLSVPs are fixed^9^ and non-deforming^10^ through time. However, numerical models suggest that the influence of sinking slabs^11^,^12^ should result in LLSVP deformation and motion^13^,^14^,^15^,^16^ over hundreds of million years. Additionally, measurements of differential splitting of SKS and SKKS seismic phases reveal anisotropy along the boundary of the African LLSVP^17^ and along the eastern boundary of the Perm Anomaly^18^, suggesting deformation is occurring on the edges of these structures. Finally, while SKS phases passing along the edge of LLSVPs suggest gradients in shear velocity that are too large to be explained by thermal variations alone^19^,^20^, how chemically distinct LLSVPs are from the rest of the mantle remains unclear^21^. Previous studies have shown that the largest-scale structure of the lower mantle results from past subduction history^11^,^12^,^13^,^15^,^16^,^22^,^23^, without quantifying the geographic match between predicted and tomographic structures or the motion of individual thermochemical structures. Although some geodynamic models^23^,^24^ produce a Perm-like anomaly linked to the African LLSVP, its tectonic origin remains to be explained.

In this study, we report paleogeographically constrained forward global mantle flow models predicting a discrete structure similar in scale and geographical location to the recently discovered Perm Anomaly, and quantify the match between predicted and seismically inferred lower mantle structure across a series of mantle flow and tomography models. In the flow models, the Perm-like anomaly forms in isolation before 150 million years ago (Myr ago), within a long-lived, ∼22,000 km-long, closed subduction network consisting of the Mongol-Okhotsk subduction zone to the west, northern Tethys subduction zone to the south, and east Asian subduction zone to the east. The models predict that the discrete Perm-like anomaly has coherently migrated westward at a rate of 1 cm year^−1^ over the last 150 Myr, which is incompatible with the hypothesis that lower mantle structures can be considered fixed and rigid over time. Because of its past mobility, the Perm Anomaly may not be linked to the Siberian Traps, but rather to the Emeishan volcanics.

## Results

### Predicted lowermost mantle temperature

**Figure 1:**
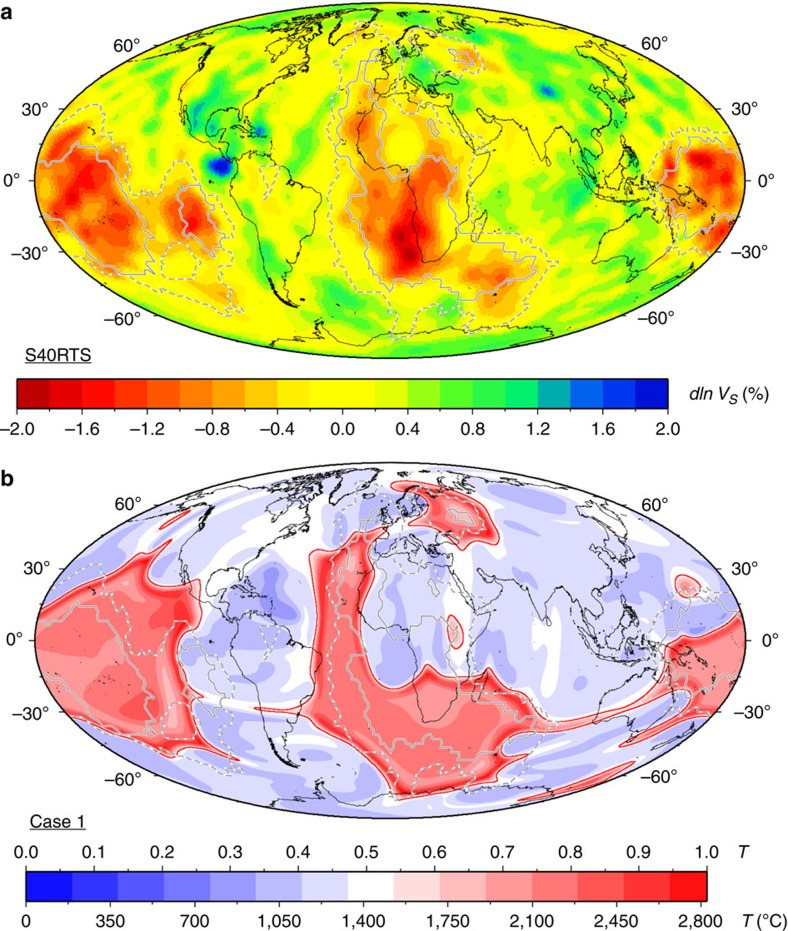
Lower mantle structure inferred from seismic tomography and predicted by a mantle flow model. (**a**) Seismic velocity anomalies at 2,677 km depth for tomography model S40RTS (ref. 27). (**b**) Predicted present-day mantle temperature at 2,677 km depth for case 1. The solid gray contour indicates a value of five, and the dashed gray contour a value of one in a vote map for tomography models^3^. Present-day coastlines are shown in black.

We address the questions raised by the discovery of the Perm Anomaly through comparison of the lowermost mantle thermal structure predicted by forward global mantle flow models constrained by tectonic reconstructions^25^ (Methods) to tomography images. In dynamic models, slabs subducting deep into the mantle deform a basal layer, initially uniform, which is either thermal or thermochemical (Methods, Table 1). In the reference model (case 1, Table 1), predicted present-day temperature ∼200 km above the core–mantle boundary (CMB) is characterized by two large high-temperature regions under Africa and the Pacific and one smaller, spatially distinct high-temperature region north-east of the African Anomaly, under eastern Europe and western Russia (Fig. 1b). For case 1, the predicted present-day CMB and surface heat flow are respectively 10.4 TW and 40.3 TW, which is consistent with constraints^26^. The spatial extent of the large high-temperature regions is in first-order agreement with the position and shape of the African and Pacific LLSVPs in individual tomography models (for example, S40RTS (ref. 27), Fig. 1a) and in a vote map of tomography models^3^ (Fig. 1), and the predicted smaller structure under eastern Europe and western Russia matches the location of the Perm Anomaly in tomography (Fig. 1). Visual comparison suggests the reference model better fits the long-wavelength shape than similar models^16^. The edges of the model LLSVPs tend to be hotter than their interior (Fig. 1), which is consistent with a plume generation zone^6^.

### Cluster analysis of mantle flow and tomography models

**Figure 2:**
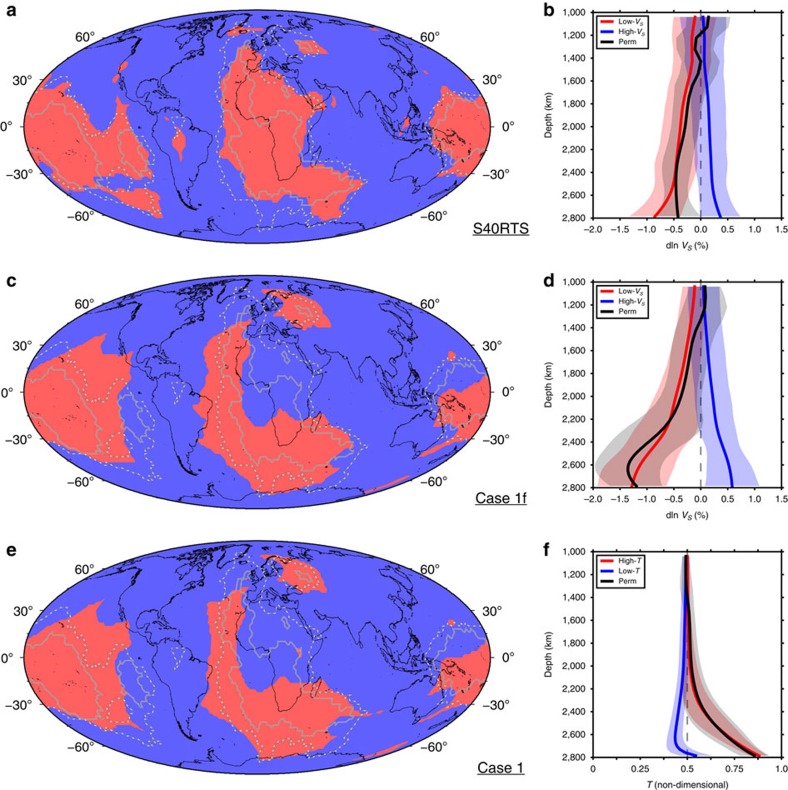
Clustering of lower mantle seismic tomography and predicted mantle temperature. (**a**) High-velocity (blue) and low-velocity (red) regions between 1,000 and 2,800 km depth for seismic tomography model S40RTS (ref. 27). (**b**) Seismic velocity profiles in high-velocity and low-velocity regions for S40RTS (ref. 27). The solid curves are the mean, and the transparent envelopes are the associated standard deviation, of the global low-velocity cluster (red), global high-velocity cluster (blue) and low-velocity cluster for the separate Perm-like anomaly (black). (**c**,**e**). Same as **a** but for seismically filtered^28^ case 1f (**c**) and case 1 (**e**). (**d**,**f**). Same as **b** but for seismically filtered^28^ case 1f (**d**) and case 1 (**f**). In **a**,**c**,**e**, the solid gray contour indicates a value of five and the dashed gray contour a value of one in a vote map for tomography models^3^. Present-day coastlines are shown in black.

To make a more quantitative comparison between lower mantle temperature predicted by global geodynamic models with seismic velocity anomalies of selected tomography images, we use cluster analysis (Methods). Cluster analysis objectively classifies a set of points into groups of points with similar variations in a given property with depth. Following Lekic *et al*.^3^ we consider two clusters and depths between 1,000 and 2,800 km (Fig. 2). For S40RTS (ref. 27), the procedure reveals a low-velocity cluster below ∼2,400 km depth, in which seismic velocity anomalies are reduced to −0.9%, and a high-velocity cluster in which seismic velocity anomalies are increased to +0.4% (Fig. 2b). For case 1f, which was seismically filtered^28^ (Methods) for direct comparison to tomography, a low-velocity and high-velocity cluster are also distinct below ∼2,400 km depth, although predicted anomalies are larger (down to −1.2% and up to +0.6%, Fig. 2d). The geographic distribution of low-velocity clusters shows two large LLSVPs and a Perm-like anomaly in both S40RTS and case 1f (Fig. 2a,c), confirming that the extent and location of predicted deep mantle structures is compatible with seismic images. Given the small influence of seismic filtering, including on the extent and location of the Perm-like anomaly (Fig. 2c,e), we do not seismically filter other cases for which the clustering procedure reveals a high-temperature and a low-temperature cluster below ∼2,400 km depth (Fig. 2f).

### Sensitivity of model success to parameters

We test the sensitivity to model parameters by considering 27 cases with varying Rayleigh number, initial model age, initial slab depth, viscosity, relative and absolute^4^,^5^,^29^ plate motions, and basal layer density (Table 1). To assess model success, we introduce a ‘Perm Score' *PS* (Table 1) that visually characterizes model clusters; the method scores whether a predicted Perm-like anomaly is present and separate from the African LLSVP (*PS*=2), present and linked to the African LLSVP (*PS*=1), or absent (*PS*=0). A Perm-like anomaly is present and separate from the African LLSVP in 15 out of 27 cases. Moreover, this Perm-like anomaly is the only isolated, small anomaly that forms in all of these 15 cases; consequently there must be a specific cause for the generation of this unique feature. The Perm-like anomaly is separate at present-day for initial model age >200 Myr ago, initial slab depth >800 km, and when absolute plate motions are based on hotspot tracks^4^,^29^ and paleomagnetic data^5^ as opposed to mapping slab remnants from seismic tomography^7^ (Table 1). These results confirm the influence of initial conditions and subduction history on model results^12^, and we verified elsewhere^23^ that models initiated with LLSVPs in the initial condition are consistent with the present-day mantle structure. No Perm-like anomaly is predicted when the Rayleigh number Ra is ten times larger or 100 times smaller than in the reference case (*Ra*=7.8 × 10^7^).

**Figure 3:**
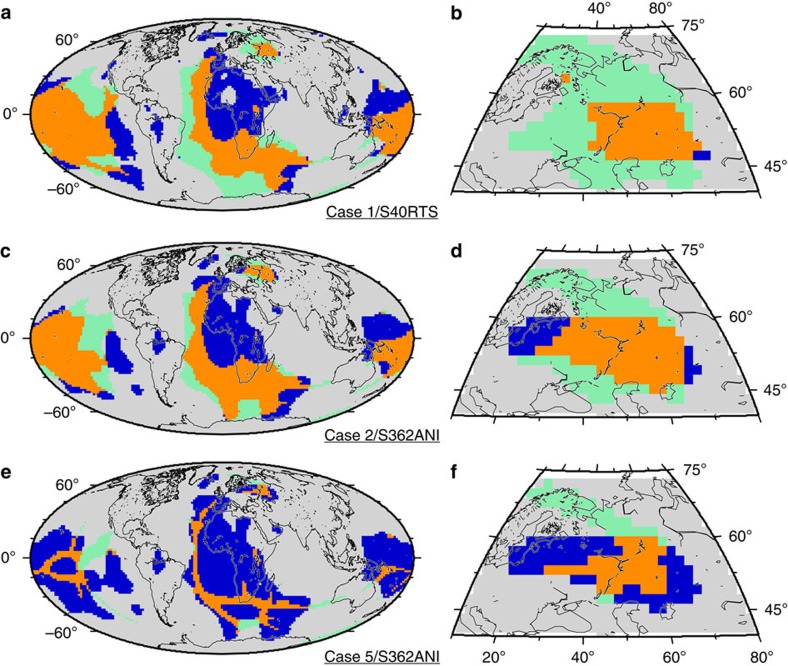
Spatial match between predicted lower mantle structure and that inferred from seismic tomography. Orange (true positive) indicates high-temperature cluster for the model and low-velocity cluster for the tomography, gray (true negative) indicates low-temperature cluster for the model and high-velocity cluster for the tomography, green (false positive) indicates high-temperature cluster for the model and high-velocity cluster for the tomography and blue (false negative) indicates low-temperature cluster for the model and low-velocity cluster for the tomography. Present-day coastlines are shown in black. Results are shown for case 1 and S40RTS (ref. 27) (**a**,**b**), case 2 and S362ANI (ref. 38) (**c**,**d**), **e**,**f**, and case 5 and S362ANI (ref. 38) (**e**,**f**). **b**,**d**,**f** show results in the region between 10°–80°E and 40°–75°N that includes the Perm Anomaly.

**Figure 4:**
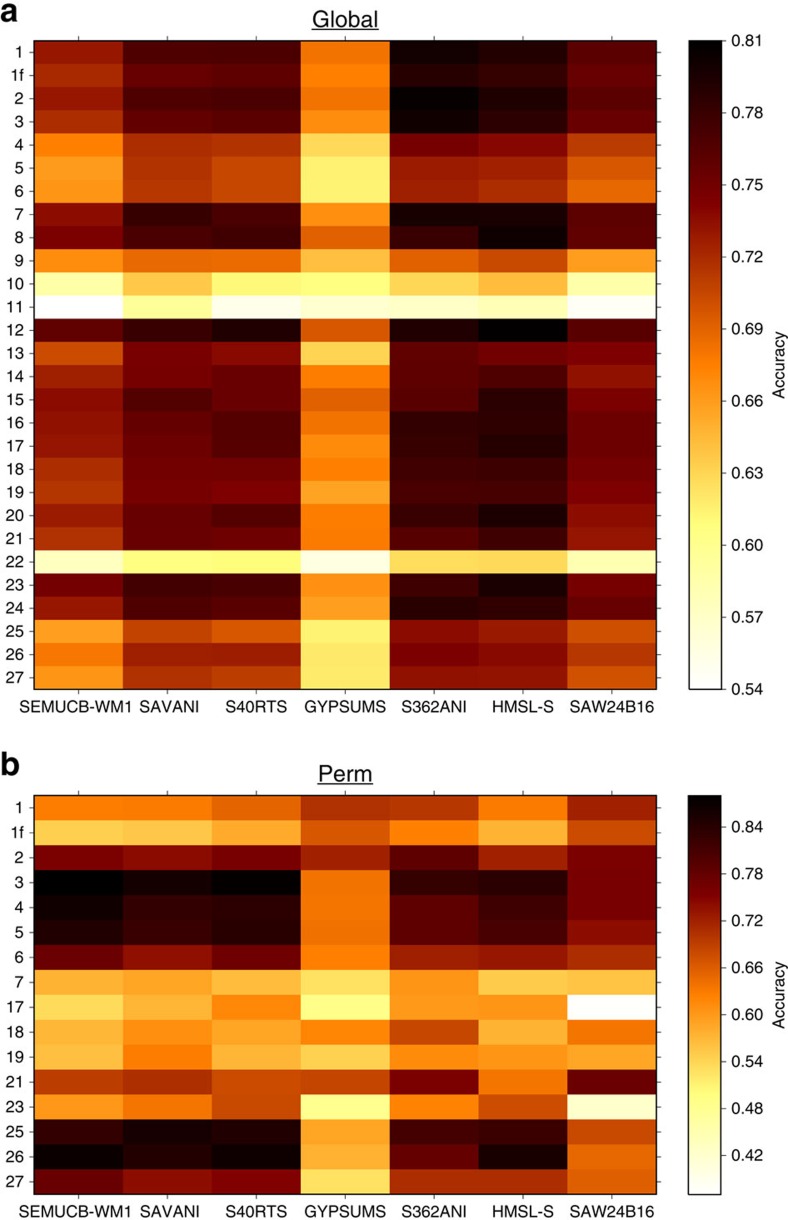
Quantitative match between predicted lower mantle structure and seismic tomography models. Accuracy (true positive area plus true negative area over total area), with which the geographical distribution of clusters of mantle temperature between 1,000 and 2,800 km depth predicted by 27 mantle flow model cases (case 1f is seismically-filtered case 1) reproduce the geographical distribution of clusters of seismic velocity anomalies between 1,000 and 2,800 km depth for seven S-wave tomography models. (**a**) global accuracy, (**b**) accuracy in the region between 10°–80°E and 40°–75°N that includes the Perm Anomaly, for cases predicting a Perm-like anomaly separate from the model African LLSVP (PS=2 in Table 1). See Table 1 for values of the global and regional accuracies averaged over the seven considered tomography models.

To assess model success beyond the prediction of a Perm-like anomaly, we calculate the accuracy with which the global geographic distribution of predicted model clusters reproduces that of tomography clusters. This accuracy, defined as the ratio of successfully predicted areas to total area (Methods, Fig. 3), is calculated for distinct mantle flow and tomography models, each of which is based on different assumptions and delivers non-unique inferences of the true pattern of mantle structure. The accuracy varies between 0.54 and 0.81 across 27 model cases and seven tomography models (Methods, Fig. 4a), and is above random (0.5) even for the least successful models. For each case, we report the average accuracy for all seven tomography models, which ranges between 0.56 and 0.76. Average accuracy decreases with decreasing initial model age, is ≤0.61 when the initial model age is 100 Myr ago or younger and when *Ra* is large (7.8 × 10^8^), and is between 0.61 and 0.71 when the basal layer is purely thermal or <2.54% chemically denser than ambient mantle (Fig. 4a; Table 1). The average accuracy is ≥0.71 for all other cases. The average accuracy for GyPSuM-S (ref. 30) (0.65) is lower than for other tomography models (between 0.69 and 0.75), which might reflect that GyPSuM-S (ref. 30) is an inversion for geodynamic and mineral physics constraints in addition to the seismic constraints used in other tomography models.

### Origin of the Perm Anomaly

**Figure 5:**
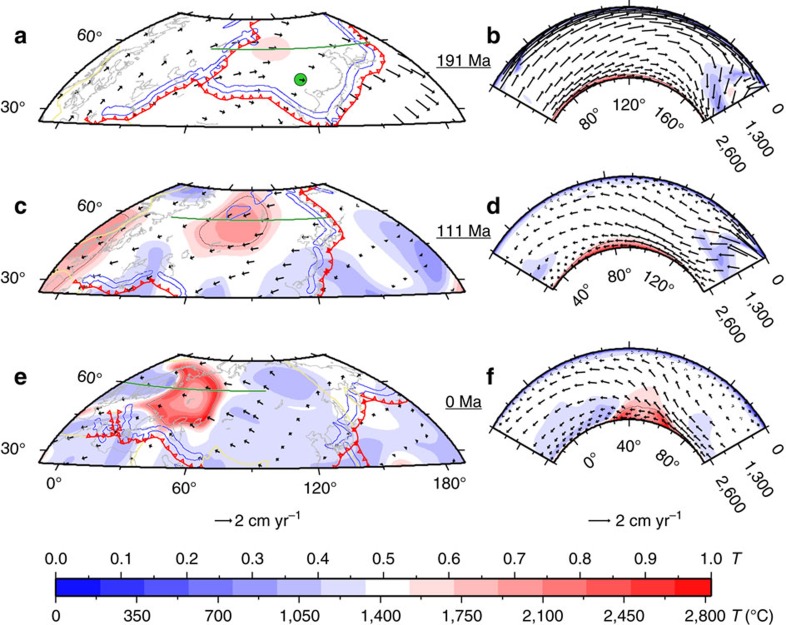
Formation of the Perm Anomaly. Predicted mantle temperature, flow and composition for case 1 at 2,677 km depth (**a**,**c**,**e**) and along cross-sections (green lines) at 60° latitude (**b**,**d**,**f**). Results are shown at 191 Myr ago (**a**,**b**), 111 Myr ago (**c**,**d**) and present-day (**e**,**f**). Reconstructed subduction locations are shown as red lines with triangles on the overriding plate, reconstructed mid-oceanic ridges and transform faults are shown as yellow lines, and reconstructed coastlines are shown in gray. The brown contours indicate 50% concentration of dense material. In **a**,**c**,**e**, the blue contours indicate subducting plates with temperature 10% lower than ambient mantle at 489 km depth. The green circle in **a** is the location of the ∼258 Myr ago Emeishan LIP, reconstructed at 191 Myr ago.

Having established that the present-day lower mantle structure is well reproduced by mantle flow, we investigate the dynamics leading to the formation of the Perm-like anomaly. The model high-temperature clusters (Fig. 2e,f) correspond to temperatures that are ∼10% higher than ambient at 2,677 km depth (Fig. 1b). Following the evolution of temperature at 2,677 km depth in the reference case (Fig. 5a,c,e) reveals that despite its present-day proximity with the African LLSVP, the incipient Perm-like anomaly formed ∼190 Myr ago centred on 100°W/60°N (Fig. 5a), between three long-lived subduction systems: Mongol-Okhotsk along Eurasia (geodynamically preferable than along Central Asia^31^) to the west, northern Tethys to the south, and east Asia to the east (Fig. 5a,b). In this tectonic scenario^32^,^33^, subduction to the west of the Perm-like anomaly ceases when the Mongol-Okhotsk Ocean closes 150 Myr ago. The Perm-like anomaly then migrates ∼1,500 km westward as pushed by descending slabs^12^,^15^,^23^ subducting under east Asia (Fig. 5c–f). The coherent translation of the discrete, Perm-like anomaly allows us to estimate an average motion rate of 1 cm year^−1^ over the last 150 Myr. This ongoing westward flow is compatible with SKS–SKKS splitting measurements revealing anisotropy with a fast east–west direction in the lowermost mantle under eastern Europe and western Russia^18^, potentially due to lattice preferred-orientation of post-perovskite^34^. Moreover, the prediction is consistent with deformation on the eastern boundary of the Perm Anomaly and the presence of high seismic velocity structures to the east of the Perm Anomaly that also reveal anisotropy in SKS–SKKS splitting measurements^18^. Together with seismic observations^18^ and previous models^13^,^14^,^15^,^16^, our results challenge the long-term fixity and rigidity of deep-mantle thermochemical structures.

## Discussion

The genesis of the Perm-like anomaly within a long-lived, closed subduction network with a perimetre ∼22,000 km (Fig. 5a) could explain why a single small LSVP^18^ is observed seismically, and why only one isolated thermochemical pile forms in our models. Although the geometry and timing of past plate boundaries is increasingly uncertain back in geological times due to decreasing amounts of preserved ocean floor^35^, this tectonic setting is unique for the last 230 Myr. We find that the Perm-like anomaly is only separate at present if slabs are inserted to depths >800 km (*PS*=2 in Table 1) in the initial condition at 230 Myr ago. This suggests that the subduction network would have been established at the latest between ∼330 and ∼280 Myr ago, depending on slab sinking rates^7^. For the reference case, the present-day Perm-like anomaly is chemically distinct (∼550 km in thickness based on 50% dense material), high temperature (∼850 km in thickness based on mantle 20% hotter than ambient) and may actively contribute to mantle upwelling (Fig. 5f). The predicted chemical anomaly is consistent with the ∼500 km thickness of the Perm Anomaly inferred from seismic images^3^, but the thermal anomaly may be overestimated in the model. In contrast to models in which the Perm-like anomaly is similar to LLSVPs (Fig. 2d), global seismic models have not reported shear-velocity anomalies in the Perm Anomaly that are as low as in the LLSVPs^3^ (Fig. 2b), although caution must be exercised as the amplitudes of seismic anomalies are often poorly constrained tomographically^36^. One possibility to explain the apparent smaller shear-velocity reduction in the Perm Anomaly^3^ (Fig. 2b) is that it could be compositionally different from the LLSVPs: decreasing the density of the basal layer results in a better match of the Perm Anomaly, but a poorer global match (Figs 3 and 4; Table 1).

Our reconstructions of past mantle flow link the formation of the Perm Anomaly to the history of subduction around east and central Asia between 230 and 150 Myr ago. The formation of the Perm Anomaly would have occurred several tens of million years after this subduction network was established, as slabs slowly sank. The Perm Anomaly appears between 220 and 80 Myr ago depending on the Rayleigh number, the initial slab depth and the initial model age (Methods, Table 1). Although tectonic uncertainties increase back in geological time, some reconstructions suggest that a closed network of subduction zones ∼20,000 km in perimeter might have been established around the Mongol-Okhotsk Ocean 410 Myr ago^37^, in which case the Perm Anomaly might have existed for much of the Phanerozoic. Structures similar to the Perm Anomaly are likely to have existed earlier in Earth's history, controlled by past subduction zone configurations.

Conceptual^6^ and geodynamic^22^,^23^,^24^ models suggest that plumes mostly rise from deep thermochemical structures to form LIPs at Earth's surface. The reconstructed location of the∼258-Myr ago Emeishan LIP falls within the network formed by the Mongol-Okhotsk, northern Tethys, and East Asia subduction zones between 230 and 150 Myr ago^33^ (Fig. 5a). In contrast, the reconstructed location of the ∼251 Myr ago Siberian Traps does not reconstruct within this subduction network between 230 and 150 Myr ago (the reconstructed location of the Siberian Traps is outside the region shown in Fig. 5a). Because the models show that the Perm anomaly originated within this subduction network, we propose that the Emeishan LIP is a possible product of the Perm Anomaly, in contrast to the Siberian Traps^3^,^8^. These competing hypotheses could be tested in future convection models including mantle plumes^23^, contrary to the models presented here, and based on tectonic reconstructions extending into the Paleozoic, but that do not assume that the Emeishan LIP originated from the Pacific LLSVP, contrary to existing reconstructions^8^,^37^.

The tectonic configuration of a ∼22,000 km network of long-lived (>80 Myr) subduction zones around east and central Asia before 150 Myr ago, unique in the last 200 Myr ago, led to the formation of a single, well-defined and isolated thermo-chemical anomaly. Numerical models reproduce this past natural experiment, and the coherent westward motion of the discrete Perm-like anomaly allows us to quantify an average motion of 1 cm year^−1^ since the Mongol-Okhotsk Ocean closed 150 Myr ago.

## Methods

### Paleogeographically constrained dynamic Earth models

We solve the equations for incompressible convection in a spherical domain with finite-elements using the code *CitcomS*^39^, modified as described in ref. 25 to progressively assimilate the velocity of tectonic plates, the age of the ocean floor and the location and polarity of subduction zones determined in one million year intervals from global plate tectonic reconstructions^32^,^33^ with continuously closing plates^40^. This semi-empirical approach ensures our computations represent Earth's imposed tectonic history, allowing us to reconstruct the history of deep mantle flow over the last 230 Myr. This approach is guided by the current intractability of computing time-dependent models of Earth's plate–mantle system with the resolution required to dynamically achieve tectonic-like features, including one-sided subduction^41^ and conserve the energy of the system simultaneously. Here we give a summary of the governing parameters and model setup that are further described in refs 25, 42.

The vigour of convection is defined by the Rayleigh number 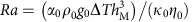, where 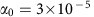
K^−1^ is the coefficient of thermal expansion, 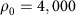
kg m^−3^ is the density, 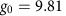
m s^−2^ is the gravity acceleration, 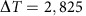
K is the temperature change across the mantle, 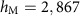
km is the thickness of the mantle, 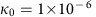
m^2^ s^−1^ is the thermal diffusivity, 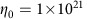
Pa s is the viscosity, and the subscript ‘0' indicates reference values. With the values listed above, 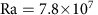. These values are varied between model cases such that Ra varies between 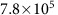 and 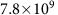 (Table 1).

We approximate the Earth's mantle as a Newtonian fluid in which viscosity varies with depth and temperature following 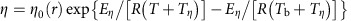, where 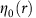 is a pre-factor defined with respect to the reference viscosity *η*_0_ for four layers: above 160 km, between 160 and 310 km depth, between 310 and 660 km depth and below 660 km depth, in the lower mantle. Values of 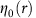 for each layer are given as comma-separated lists in Table 1, where ‘10→100' indicates that the reference viscosity linearly increases with depth from 10 to 100 throughout the lower mantle, and 0.1/1 indicates that the reference viscosity of the asthenosphere is 0.1 under oceanic plates and 1 under continental plates. *E*_*η*_ is the activation energy taken as 100 kJ mol^−1^ in the upper mantle and 30 kJ mol^−1^ in the lower mantle, *R*=8.31 J mol^−1^ K^−1^ is the universal gas constant, *T* is the dimensional temperature, 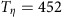
K is a temperature offset and 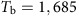
K is the ambient mantle temperature. The activation energy and temperature offset are chosen to limit variations in viscosity to three orders of magnitude across the range of temperatures without imposing a yield stress. Such lateral viscosity contrasts are lower than expected to occur within the solid Earth^41^, but they can be computed with a resolution that allows us to compute time-dependent mantle flow models. A phase change *Γ* at 660 km depth, as described in Flament *et al*.^42^ is considered in some model cases (Table 1).

For the initial condition and progressive data assimilation, the thickness and temperature of the lithosphere are derived using a half-space cooling model and the synthetic age of the ocean floor^25^, and simplified tectonothermal ages for the continental lithosphere^42^. The global thermal structure of slabs is constructed from the location of subduction zones and from the age of the ocean floor^25^. The global thermal structure of the lithosphere and of subducting slabs is assimilated in the dynamic models in 1 Myr increments, to 350 km depth at subduction zones^25^. In the initial condition, subduction zones are inserted in the mantle assuming a descent rate of 3 cm year^−1^ in the upper mantle and 1.2 cm year^−1^ in the lower mantle^7^. Subduction zones that appear during the model are progressively inserted to 350 km depth based on the age of subduction initiation and on the plate convergence rate.

The initial condition, derived from the tectonic reconstruction at 230 Myr ago, includes a basal layer just above CMB, which is either purely thermal or thermochemical (Table 1). The layer is 113 km thick, which represents 2% of the volume of the mantle, consistent with the seismically inferred value^2^. The composition of that layer is modeled using tracers^42^ and its chemical density 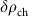
is varied between +0.85% and +4.24% (Table 1) by changing the buoyancy ratio 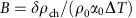 between 0.1 and 0.5, with increment 0.1. Slabs are initially inserted down to a depth of either 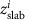 (varied between 425 km and 1,750 km; Table 1), or to the depth derived from their initiation age and sinking rates if that depth is shallower than 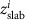, with a dip of 45° down to 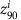 and a dip of 90° below 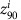 (either 425 or 660 km; Table 1). Slabs are initially twice as thick in the lower mantle compared with their thickness in the upper mantle, to account for advective thickening in the more viscous lower mantle.

The model consists of 129 × 129 × 65 × 12 ≈13 × 10^6^ nodes, which with a radial mesh refinement that gives average resolutions of∼50 × 50 × 15 km at the surface,∼28 × 28 × 27 km at the CMB, and∼40 × 40 × 100 km in the mid-mantle.

We investigate the influence of relative and absolute plate motions across five global tectonic reconstructions (*R* in Table 1). Reconstruction A, which uses the absolute plate motions of ONeill *et al*.^4^ between 0 and 100 Myr ago and that of Steinberger and Torsvik^5^ before 100 Myr ago, is described in Seton *et al*.^32^ and extended from the last 200 Myr ago to the last 230 Myr ago^33^. Continuously-closing plate polygons^40^ and ages of the ocean floor^32^,^33^, necessary to assimilate plate reconstructions in mantle flow models with the method described in Bower *et al*.^25^ are available to us back to 230 Myr ago. Reconstruction B includes changes to relative plate motions in the Arctic region^33^ and uses the absolute plate motions of Torsvik *et al*.^29^ between 0 and 70 Myr ago and that of Steinberger and Torsvik^5^ before 105 Myr ago, with interpolation between the two absolute plate motion models between 70 and 105 Myr ago. Reconstruction C includes changes to relative plate motions in Southeast Asia^33^ compared with reconstruction B. Reconstruction D includes changes to relative plate motions in the western Tethys^33^ compared with reconstruction C. Reconstruction E is based on the absolute plate motions of van der Meer *et al*.^7^ and on the same relative plate motions as reconstruction D. Reconstruction F uses the same absolute plate motion model as reconstruction B, and relative plate motions as described in Muller *et al*.^33^

### Cluster analysis of lower mantle structure

We use cluster analysis to objectively classify a set of points on the surface into groups of points with similar variations in a given property with depth. For each flow model case (or tomography model), temperature (or seismic velocity) profiles are treated as 196,596 independent vectors of 31 coordinates specifying the temperatures (or seismic velocities) sampled at 31 depths between 1,000 and 2,800 km^3^, with an average resolution of 58 km. Each vector corresponds to equally-spaced locations on Earth's surface (average distance ∼0.45°). The vectors are grouped into two clusters using *k*-means clustering^43^, a procedure that keeps the variance in squared Euclidean distance between vectors small within each cluster. We use the scientific Python implementation of the *k*-means algorithm (http://docs.scipy.org/doc/scipy/reference/generated/scipy.cluster.vq.kmeans2.html). The average and standard deviation of temperature profiles for each cluster are shown in Fig. 2 for the reference case, along with the average and standard deviation of temperature profiles for the high-temperature cluster in the Perm region.

### Seismic filtering

We seismically filter our reference case 1 following Ritsema *et al*.^28^ to verify if lower mantle features apparent in the model temperature field would be resolved by global tomography^27^. We consider that both temperature and composition variations cause variations in shear velocity. For the thermal contribution, we determine wave speed variations (d*V*_S_) scaling departures from average temperature at each depth (d*T*) using d*V*_S_/d*T*=−7.0 × 10^−5^ km s^−1^ K^−1^ (ref. 44). For the chemical contribution, we determine wave speed variations (d*V*_S_) from variations in the composition field at each depth (d*C*) using d*V*_S_/d*C*=4 × 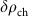 (ref. 45), where 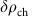 is the density of the chemically distinct basal layer. Thermal and compositional contributions to wave speed variations are jointly considered following d*V*_s_=d*V*_s_/d*T* × d*T*+*f*_c_ × d*V*_s_/d*C* × d*C,* where *f*_c_=0.05 is the contribution of composition to total wave speed anomalies.

### Quantification of model success

We quantify how well the clusters obtained for each model case reproduce the global geographic distribution of clusters obtained for global S-wave tomography models^27^,^30^,^38^,^46^,^47^,^48^,^49^ by computing the accuracy 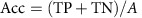, where TP is the area of true positives, TN the area of true negatives and *A* the total area. Three examples of the spatial distribution of true positives, true negatives, false positives and false negatives with respect to tomography models are shown in Fig. 3. The accuracy is computed for each model case, both globally and in the region between 10°–80°E and 40°–75°N that includes the Perm Anomaly, against seven global S-wave tomography models (Fig. 4): SAW24B16 (ref. 46), HMSL-S (ref. 47), S362ANI (ref. 38), GyPSuM-S (ref. 30), S40RTS (ref. 27), Savani (ref. 48), SEMUCB-WM1 (ref. 49). Values of the global and regional accuracies averaged over the seven tomography models are reported in Table 1.

### Age of the Perm-like anomaly

We report the age *a*_p_ from which the separate Perm-like anomaly is >190 km thick (based on a mantle temperature 10% higher than ambient, and with model output every∼10 Myr) in Table 1.

### Data availability

Maps of the geographic distribution of tomography and flow model clusters reported are available at https://www.earthbyte.org/originevolution-perm-anomaly/. The computer code that supports the findings of this study is available from the corresponding author upon reasonable request.

## Additional information

**How to cite this article:** Flament, N. *et al*. Origin and evolution of the deep thermochemical structure beneath Eurasia. *Nat. Commun.* **8,** 14164 doi: 10.1038/ncomms14164 (2017).

**Publisher's note:** Springer Nature remains neutral with regard to jurisdictional claims in published maps and institutional affiliations.

## Figures and Tables

**Table 1:** Input parameters and output metrics of model cases.

**Case**	**Input**								**Output**			
	***Ra***	***η*** 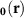	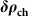	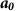 **(Myr ago)**	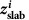 **(km)**	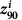 **(km)**	***Γ***	***R***	***PS***	***Acc***_**G**_	***Acc***_**P**_	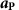 **(Myr ago)**
1	7.8E+07	1,1,1,10→100	4.24%	230	1,400	425	N	C	2	0.76	0.67	191
1f	7.8E+07	1,1,1,10→100	4.24%	230	1,400	425	N	C	2	0.75	0.60	191
2	7.8E+07	1,1,1,10→100	**3.39%**	230	1,400	425	N	C	2	0.76	0.75	190
3	7.8E+07	1,1,1,10→100	**2.54%**	230	1,400	425	N	C	2	0.75	0.81	190
4	7.8E+07	1,1,1,10→100	**1.70%**	230	1,400	425	N	C	2	0.70	0.79	190
5	7.8E+07	1,1,1,10→100	**0.85%**	230	1,400	425	N	C	2	0.69	0.78	190
6	7.8E+07	1,1,1,10→100	**N/A**	230	1,400	425	N	C	2	0.69	0.72	190
7	7.8E+07	1,1,1,10→100	4.24%	230	1,400	425	N	**F**	2	0.76	0.57	190
8	7.8E+07	1,1,1,10→100	4.24%	**200**	1,400	425	N	**F**	1	0.76	N/A	150
9	7.8E+07	1,1,1,10→100	4.24%	**150**	1,400	425	N	**F**	1	0.68	N/A	90
10	7.8E+07	1,1,1,10→100	4.24%	**100**	1,400	425	N	**F**	1	0.61	N/A	19
11	7.8E+07	1,1,1,10→100	4.24%	**50**	1,400	425	N	**F**	1	0.56	N/A	N/A
12	7.8E+07	1,1,1,10→100	4.24%	230	**425**	425	N	**F**	1	0.77	N/A	129
13	7.8E+07	1,1,1,10→100	**1.70%**	230	**425**	425	N	**F**	1	0.72	N/A	139
14	7.8E+07	**1,1,1,100**	4.24%	230	**425**	425	N	C	1	0.74	N/A	120
15	7.8E+07	**1,1,10,100**	4.24%	300*	**500**	425	**Y**	**D**	1	0.75	N/A	220
16	7.8E+07	**1,1,1,100**	4.24%	230	**800**	425	N	C	1	0.75	N/A	141
17	7.8E+07	**1,0.1,1,100**	4.24%	230	**1,100**	425	N	C	2	0.75	0.54	190
18	7.8E+07	**1,1,1,100**	4.24%	230	**1,200**	**660**	N	**B**	2	0.74	0.61	170
19	7.8E+07	**1,1,1,100**	4.24%	230	**1,750**	**660**	N	**B**	2	0.73	0.59	190
20	**7.8E+05**	**1,1,1,100**	4.24%	230	**1,200**	425	N	C	0	0.75	N/A	N/A
21	**1.3E+07**	1,1,1,10→100	4.24%	230	**1,200**	**660**	N	C	2	0.74	0.70	79
22	**7.8E+08**	1,1,1,10→100	4.24%	230	**1,200**	**660**	N	C	0	0.60	N/A	N/A
23	7.8E+07	**1,0.1/1,1,100**	4.24%	230	1,400	425	N	C	2	0.75	0.59	190
24	7.8E+07	**1,0.1,1,100**	4.24%	230	1,400	425	N	**E**	1	0.75	N/A	190
25	7.8E+07	**1,1,1,10**→**100**	**1.70%**	230	**1,200**	425	N	**A**	2	0.69	0.78	200
26	7.8E+07	**1,0.1,1,10**→**100**	**1.70%**	230	**1,200**	**660**	**Y**	**B**	2	0.71	0.78	190
27	7.8E+07	**1,1,1,100**	**N/A**	230	**1,200**	425	N	C	2	0.70	0.70	189

*Ra* is the Rayleigh number, *η*_0_(*r*) is a pre-factor defined with respect to the reference viscosity *η*_0_ for four layers: above 160 km, between 160 and 310 km depth, between 310 and 660 km depth and below 660 km depth (where 10→100 indicates that the reference viscosity linearly increases with depth across the lower mantle, and 0.1/1 indicates that the reference viscosity of the asthenosphere is 0.1 under oceanic plates and 1 under continental plates), *δρ*_ch_ is the chemical density of the basal layer, derived from the buoyancy ratio *B*, *a*_0_ is the age at which the model starts (* indicates that the same boundary conditions are repeated between 300 and 230 Myr ago), 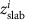 is the initial slab depth, 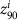 is the depth from which the initial dip angle of slabs changes from 45° to 90°, *Γ* indicates whether a phase change is considered at 660 km depth, *R* is the reconstruction, *PS* is a score indicating with value 0 if a Perm-like anomaly is not predicted, 1, if it is, and 2 if it is predicted and separate from the African LLSVP, *Acc*_G_ is the global accuracy, *Acc*_P_ is the accuracy in the Perm region, and *a*_p_ is the age from which the Perm-like anomaly exists. Parameters in bold are different from the reference case. See Methods for more details.
